# Predictors and one-year outcomes of patients with delayed graft function after deceased donor kidney transplantation

**DOI:** 10.1186/s12882-020-02181-1

**Published:** 2020-12-04

**Authors:** Rao Chen, Haifeng Wang, Lei Song, Jianfei Hou, Jiawei Peng, Helong Dai, Longkai Peng

**Affiliations:** 1 Department of Kidney Transplantation, The Second Xiangya Hospital of Central South University, Changsha, Hunan 410011 China; 2 Clinical Research Center for Organ Transplantation in Hunan Province, Changsha, Hunan 410011 China; 3 Clinical Immunology Center, Central South University, Changsha, Hunan 410011 China

**Keywords:** Delayed graft function, Predictors, Nomogram, Deceased donation, Graft survival

## Abstract

**Background:**

Delayed graft function (DGF) is closely associated with the use of marginal donated kidneys due to deficits during transplantation and in recipients. We aimed to predict the incidence of DGF and evaluate its effect on graft survival.

**Methods:**

This retrospective study on kidney transplantation was conducted from January 1, 2018, to December 31, 2019, at the Second Xiangya Hospital of Central South University. We classified recipients whose operations were performed in different years into training and validation cohorts and used data from the training cohort to analyze predictors of DGF. A nomogram was then constructed to predict the likelihood of DGF based on these predictors.

**Results:**

The incidence rate of DGF was 16.92%. Binary logistic regression analysis showed correlations between the incidence of DGF and cold ischemic time (CIT), warm ischemic time (WIT), terminal serum creatine (Scr) concentration, duration of pretransplant dialysis, primary cause of donor death, and usage of LifePort. The internal accuracy of the nomogram was 83.12%. One-year graft survival rates were 93.59 and 99.74%, respectively, for the groups with and without DGF (*P* < 0.05).

**Conclusion:**

The nomogram established in this study showed good accuracy in predicting DGF after deceased donor kidney transplantation; additionally, DGF decreased one-year graft survival.

**Supplementary Information:**

The online version contains supplementary material available at 10.1186/s12882-020-02181-1.

## Background

Kidney transplantation has been successful and has saved numerous lives since the 1960s. Compared to regular kidney dialysis, kidney transplantation results in better patient quality of life and longer survival [1]. Deceased donation (DD), including donation after brain death (DBD), donation after cardiac death (DCD), and donation after brain death awaiting cardiac death (DBCD), has been promoted in recent years throughout China, allowing an increasing numbers of patients to recover from uremia. However, DGF is one of the most common postoperative complications [2, 3], the incidence of which varies from 5 to 50% in DD kidney transplantation. There is a scarcity of donated kidneys, and the use of marginal kidneys has dramatically increased the incidence of DGF, which is not only a risk factor for acute rejection but is also associated with poor long-term survival of the graft [4, 5]. Some centers believe that DGF in transplantation is a specific manifestation of acute tubular necrosis (ATN) during the procedure [6]. In general, renal graft function recovery includes the following types: immediate graft function (IGF), slow graft function (SGF), and DGF.

Various potential factors affect the rate of DGF, including the induction strategy, donor criteria, and surgical process, among others. Our center utilizes expanded criteria donor (ECD) kidneys to address the shortage, which has also been recognized by the Organ Procurement and Transplantation Network (OPTN) in recent years [7]. Kidney transplantations from pediatric donors were shown to be safe in a recent study [8]. Our center has also adopted partial graft from young children (≤ 12 years) [9]; despite the difficulty of surgery, the youngest donor in this study was only 5 months old. Considering the contradiction between marginal kidneys and DGF, this retrospective study was conducted based on the medical records of our center to investigate pretransplant risk factors for DGF. This study also established a visual scoring system (nomogram) model for predicting clinical outcomes regarding the incidence of DGF. Finally, we compared graft survival between DGF and non-DGF groups.

## Methods

### Patients

Data for consecutive patients who had undergone kidney transplantation surgery were collected at the Second Xiangya Hospital of Central South University. The study was approved by the Ethics Committee of the Second Xiangya Hospital of Central South University, which waived the requirement for informed consent due to the retrospective design. The inclusion criteria were (1) grafts obtained from DD, (2) surgery performed as single-kidney transplantation, (3) and patients with complete data. Patients with a history of kidney transplantation or who had received double-kidney transplantations or grafts from living donors were excluded. The term ECD was used to classify subsets of all DDs over 60 years and DDs aged 50–59 years with at least two of the following characteristics: history of hypertension, serum creatinine (Scr) concentration above 1.5 mg/dL, and DCD [10]. Among marginal kidneys, if possible, we used LifePort Kidney Transporters for hypothermic machine perfusion (HPM) to potentially minimize preservation injury. DGF was defined as the requirement for dialysis within the first week after transplantation [11, 12]. We further classified patients into those who did or did not experience DGF.

Eligible patients who underwent surgery between January 1 and December 31, 2018, were included in the training cohort for the development of the nomogram; those who underwent surgery between January 1 and December 31, 2019, were included in the validation cohort.

### LifePort

Hemodynamic instability will destroy the utility of organs if the kidney has a long WIT or hypotension, which may cause blood clots, affecting the appearance and texture of the kidney. When we found a kidney that was reddish in color or slightly tough in texture, we used LifePort to evaluate and improve its quality before making a decision. A flow of > 80 mL/min was the screening criterion of transplantation qualification [13]. We chose 30 mmHg as the initial pressure and 2 h as the average perfusion time If the average terminal flow was less than 80 mL/min, the kidney was discarded. Other kidneys with good appearance and texture were not subjected to HMP (LifePort), we classify LifePort as “No” in Table 2.

### Immunity induction therapy

Antithymocyte globulin (ATG) or interleukin 2 (IL-2) receptor antibody blockers with steroids were used as induction therapy according to the surgeons’ experience. As some surgeons only administered steroids and drugs for cases with a significant risk of infection, we classify immunity induction as “Yes” or “No” in Table 2.

### Relevant variables

DGF-related factors included donor and recipient factors. We collected donor data, including age, sex, height, weight, BMI, blood type, CIT, WIT, primary cause of death, terminal urine volume before organ harvest, terminal Scr concentration before organ harvest, intensive care unit (ICU) duration, hypotension history, cardiac arrest time, donation type, history of hypertension and diabetes, history of cardiopulmonary resuscitation (CPR), history of hepatitis C virus (HCV) and usage of LifePort. The kidney donor profile index (KDPI), as an influencing factor of graft survival and DGF in some centers [14–16], was also incorporated into our study.

Recipient indicators included sex, age, height, weight, BMI, preoperative Scr concentration, number of human leukocyte antigen (HLA) mismatches, preoperative plasma renin activity (PRA) level, dialysis type, pretransplant dialysis duration, surgery duration, and immunity induction history.

## Statistics

Statistical analyses were conducted using IBM SPSS Statistics for Windows, version 23.0. For univariate analysis, continuous variables were compared using unpaired, two-tailed t-tests; categorical variables were compared by χ2 or Fisher exact tests when data were sparse. Binary logistic regression was performed to assess the impact of significant DGF-related factors in univariate analysis. *P* values < 0.05 were considered statistically significant. R software version 4.0 were used to graphically depict the impact of significant risk factors identified in the binary logistic regression and to develop the nomogram. We used the parameters of the odds ratios and β of the predictors to assess the likelihood of DGF.

The internal predictive accuracy of the model was evaluated in the training cohort using the area under the receiver operating characteristic curve (AUC) derived from 10-fold cross-validation by the least absolute shrinkage and selection operator (LASSO). The validation cohort was used to perform external validation by logistic analysis and calibration curves were plotted to examine the calibration of the nomogram, accompanied by Hosmer–Lemeshow tests. One-year graft survival curves were generated using the Kaplan–Meier method and compared using log-rank tests.

## Results

### Patient screening

During the study period, 858 consecutive patients underwent kidney transplantation. Of these, 721 patients who met the inclusion criteria were enrolled, with 461 and 260 patients assigned to the training and validation cohorts, respectively (Figure S1 in the Supplement). The training cohort included 78 cases with DGF and 383 cases without DGF; the validation cohort included 23 cases with DGF and 237 cases without DGF.

### Univariate analysis of the training cohort

With regard to the training cohort data, the proportion of ECD was 20.82%, and the incidence of DGF was 16.92%; ECD showed no statistical significance with the occurrence of DGF(*P* > 0.05). The recipients tended to be younger with just five people older than 60 years old; the median age was 37 years old. The percentages of young child donors (≤ 12 years) and aged donor (≥ 60 years) were 13.88 and 17.15%, respectively. The remaining significant (*P* < 0.05) variables were fitted to a binary logistic regression model. Continuous variables (Table 1) are presented as means and standard deviations (SDs) and categorical data (Table 2) as proportions and percentages.

**Table 1:** Characteristics of each continuous variable

Variables	Mean ± SD
Donor demographics	
Age (years)	40.91 ± 19.77
Donor clinical characteristics	
KDPI (%)	57 ± 34
Weight (Kg)	59.35 ± 19.80
BMI (Kg/ m^2^)	22.8 ± 4.29
CIT (hours)	12.35 ± 3.86
WIT (minutes)	1.94 ± 2.08
Terminal Scr (mg/dL)	0.94 ± 0.56
Cardiac arrest time (minutes)	3.25 ± 12.26
Terminal urine volume (mL/h)	173.43 ± 191.23
Duration of ICU	7.26 ± 13.6
Recipient demographics	
Age (years)	37.83 ± 10.50
Recipient clinical characteristics	
Weight (Kg)	59.72 ± 12.44
BMI (Kg/m^2^)	22.02 ± 3.66
Pretransplant dialysis duration (months)	23.02 ± 25.22
Pretransplant Scr (mg/dL)	11.64 ± 3.95
HLA mismatches	4.16 ± 1.38

**Table 2:** Characteristics of each categorical variable

Variables	With DGF (*n* = 78)	Without DGF (*n* = 383)
Donor demographics		
Gender, n (%)		
Male	59 (75.64)	262 (68.41)
Female	19 (24.36)	121 (31.59)
Age, n (%)		
Young children (≤ 12 years)	10 (12.82)	12 (15.38)
Adolescents and adults (13–59 years)	56 (71.79)	262 (68.41)
The aged (≥ 60 years)	12 (15.38)	67 (17.49)
Donor clinical characteristics		
Donor type, n (%)		
DBD	56 (71.79)	318 (83.03)
DCD	21 (26.92)	54 (14.10)
DBCD	1 (1.28)	1 (0.26)
History of hypertension, n (%)		
Yes	28 (35.90)	123 (32.11)
No	48 (61.54)	240 (62.66)
Unknown	2 (2.56)	20 (5.22)
History of diabetes, n (%)		
Yes	9 (11.54)	38 (9.92)
No	55 (70.51)	315 (82.25)
Unknown	14 (17.95)	30 (7.83)
History of CPR, n (%)		
Yes	20 (25.64)	59 (15.40)
No	58 (74.36)	324 (84.60)
Primary cause of death, n (%)		
Head trauma	2 (2.57)	158 (41.25)
Stroke	58 (74.36)	161 (42.03)
Other	18 (23.08)	64 (16.71)
History of hypotension, n (%)		
Yes	54 (69.23)	214 (55.87)
No	24 (30.77)	169 (44.13)
History of HCV, n (%)		
Yes	2 (2.56)	4 (1.04)
No	76 (97.44)	379 (98.96)
LifePort, n (%)		
Yes	5 (6.41)	4 (1.04)
No	73 (93.59)	379 (98.96)
Recipient demographics		
Gender, n (%)		
Male	59 (75.64)	262 (68.41)
Female	19 (24.36)	121 (31.59)
Recipient clinical characteristics		
Primary disease for renal failure, n (%)		
Diabetes	3 (3.85)	7 (1.83)
Hypertension	13 (16.67)	47 (12.27)
Purpura nephritis	0 (0)	3 (0.78)
Urologic obstruction	1 (1.28)	1 (0.26)
Polycystic kidney	4 (5.13)	6 (1.57)
Vasculitis	0 (0)	9 (2.35)
Other	58 (74.36)	313 (81.72)
PRA level, n (%)		
Positive	3 (3.85)	25 (6.53)
Negative	75 (96.15)	358 (93.47)
Immunity Induction, n (%)		
Yes	63 (80.77)	312 (81.46)
No	15 (19.23)	71 (18.54)

### Binary logistic regression analysis

Risk factors with statistical significance in the univariate analysis were sequentially examined using binary logistic regression analysis. However, only six factors correlated significantly with DGF occurrence, including CIT, WIT, donor terminal Scr, usage of LifePort, primary cause of death, and recipient duration of pretransplant dialysis (Table 3).

**Table 3:** The results of binary logistic regression analysis

Variables	β	OR(95%CI)
**Donor factors**		
CIT (hours)	0.075	1.078 (1.001–1.161)
WIT (minutes)	0.086	1.303 (1.101–1.045)
Terminal Scr (mg/dL)	0.641	1.899 (1.206–2.989)
Cardiac arrest time (minutes)	0.015	1.015 (0.986–1.145)
Donation type		
DBD	−0.527	0.591 (0.026–13.651)
DBCD	−0.495	0.610 (0.258–1.141)
CPR history		
No	0.048	1.049 (0.417–2.640)
History of diabetes		
No	−0.461	0.631 (0.242–1.706)
Unknown	0.285	1.330 (0.403–4.384)
Primary cause of death		
Head trauma	−2.891	0.056 (0.012–0.257)
Stroke	0.105	1.375 (0.626–3.019)
History of hypotension		
No	−0.511	0.600 (0.313–1.149)
LifePort		
No	−2.130	0.119 (0.021–0.666)
**Recipient factors**		
Pretransplant dialysis duration (months)	0.011	1.012 (1.000–1.023)

As to categorical variables, the reference categories above are DCD, with CPR history, with diabetes, other, with hypotension, with usage of LifePort, respectively

*β* coefficient from binary logistic regression model, *OR* odds ratio, *CI* confidence interval

### DGF risk nomogram

**Fig. 1:**
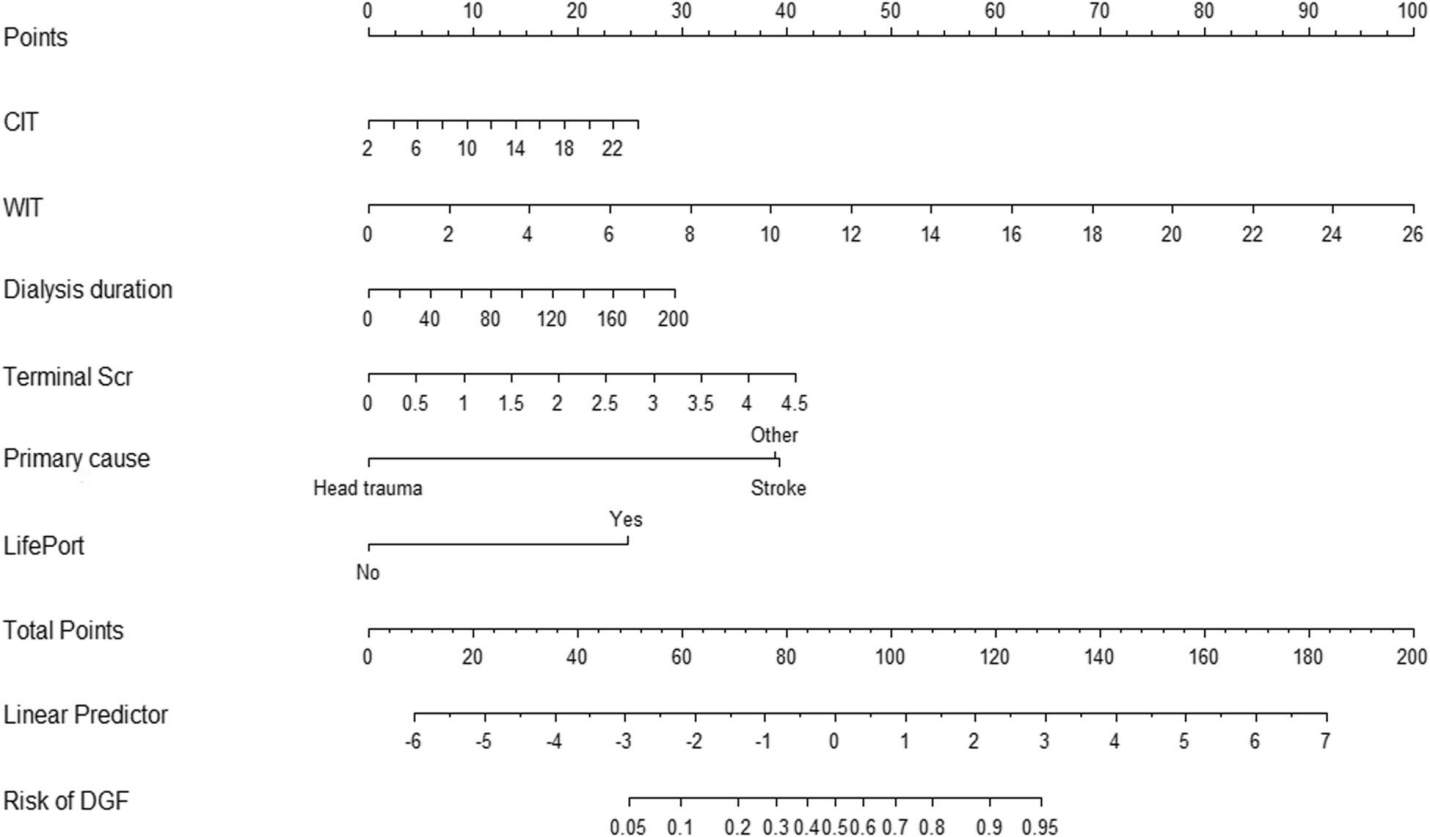
Nomogram for predicting the incidence of DGF. The statistically significant factors of binary logistical regression are shown in the nomogram, including CIT, WIT, pretransplant duration of dialysis, terminal Scr, primary cause of death and LifePort, successively. The risk of DGF was calculated, with the 95% CI shown. The code of creating the nomogram by R software is provided in Supplement 2. The weight and score of each predictor are shown in Supplement 3

To visualize the results of binary logistic regression, we used all data for the six significant variables to develop a nomogram model, as shown in Fig. 1. One hundred points were assigned to the most effective factor (WIT) in the nomogram, followed by donor terminal Scr (41 points) and primary cause of death (39 points). Usage of LifePort had the least effect (25 points); CIT was assigned 26 points. Among recipient factors, pretransplant dialysis duration was assigned 29 points. A total score of 89 corresponded to a DGF incidence of 50%.

### Model validation

**Fig. 2:**
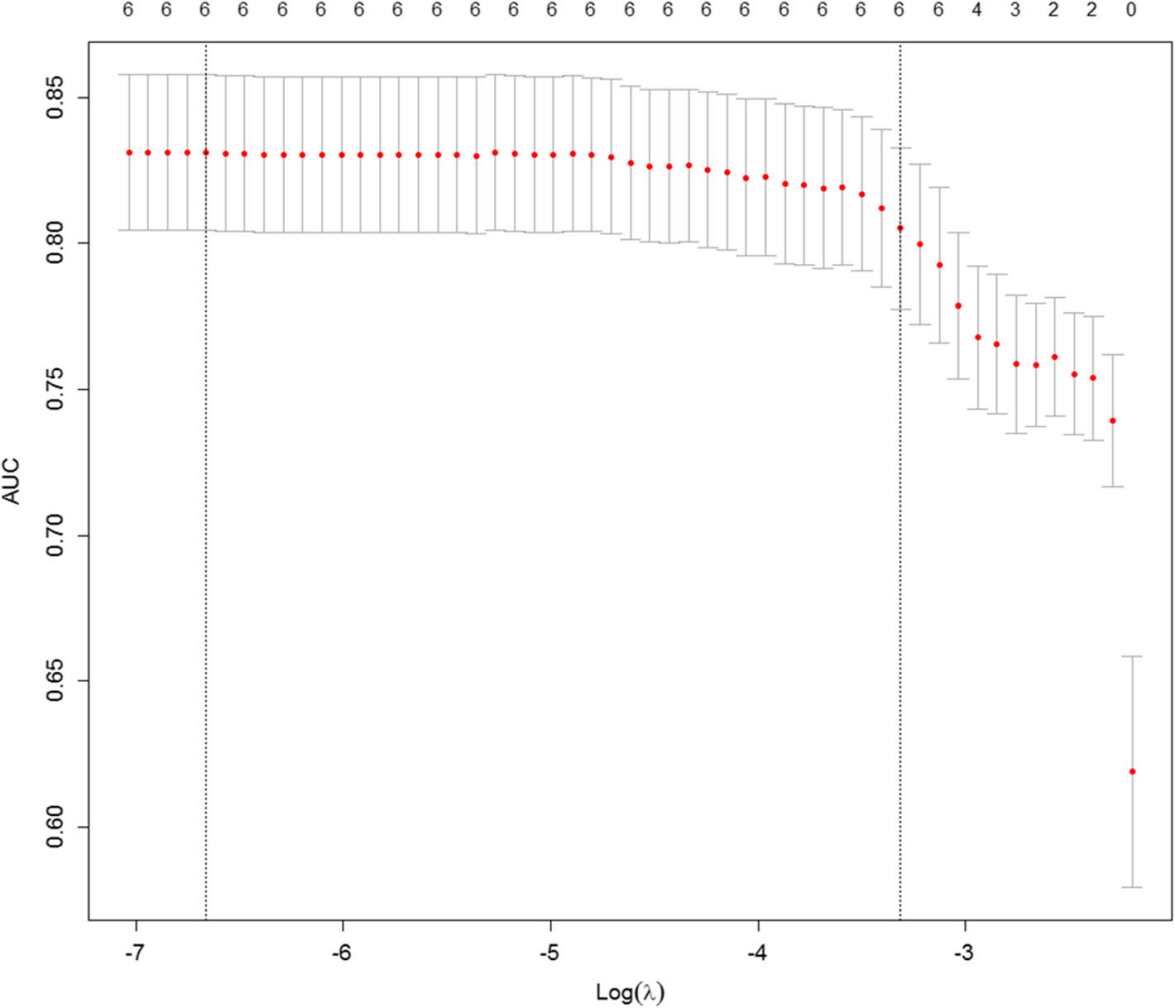
Internal validation: AUC plot by LASSO. With the log (lambda) value corresponding to the minimum mean-squared error value, the AUC value produced from 10-fold cross-validation by LASSO was 83.12%

The nomogram model was internally validated using a cross-validation method through a LASSO binary logistic regression model (Fig. 2). The LASSO model used 10-fold cross-validation via minimum criteria. The AUC was plotted versus log (lambda); the AUC value of 83.12% indicated that the model was accurate.

**Fig. 3:**
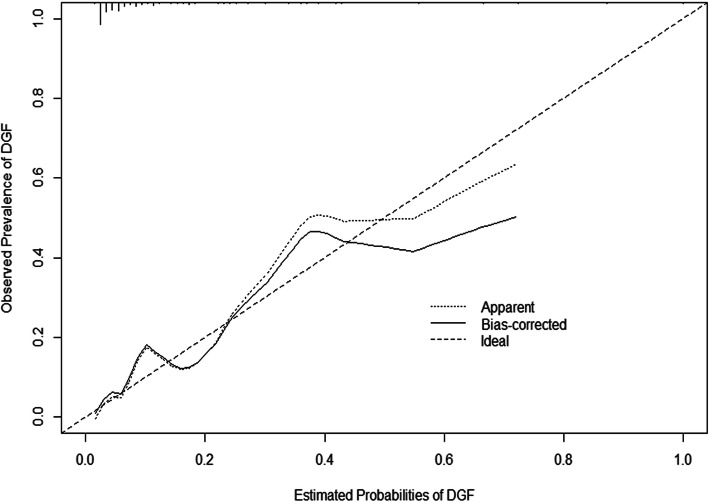
Calibration plot of the validation cohort. The x-axis represents the predicted DGF risk; the y-axis represents the actual DGF rate. The diagonal dashed line represents a perfect prediction by an ideal model and the dotted line the performance of the nomogram; the plot shows good agreement between the predicted probabilities and the observed prevalence of DGF

External validation was performed with the validation cohort using the logistic regression formula from the training cohort. The statistic (*P* = 0.636) from the Hosmer–Lemeshow test showed good calibration between predicted and observed DGF (Fig. 3). The relatively corrected C-index derived from bootstrapping validation (1000 bootstrap resamples) for the estimation of DGF risk was 0.846 (95% confidence interval [CI], 0.765 to 0.926).

### One-year graft survival follow-up

**Fig. 4:**
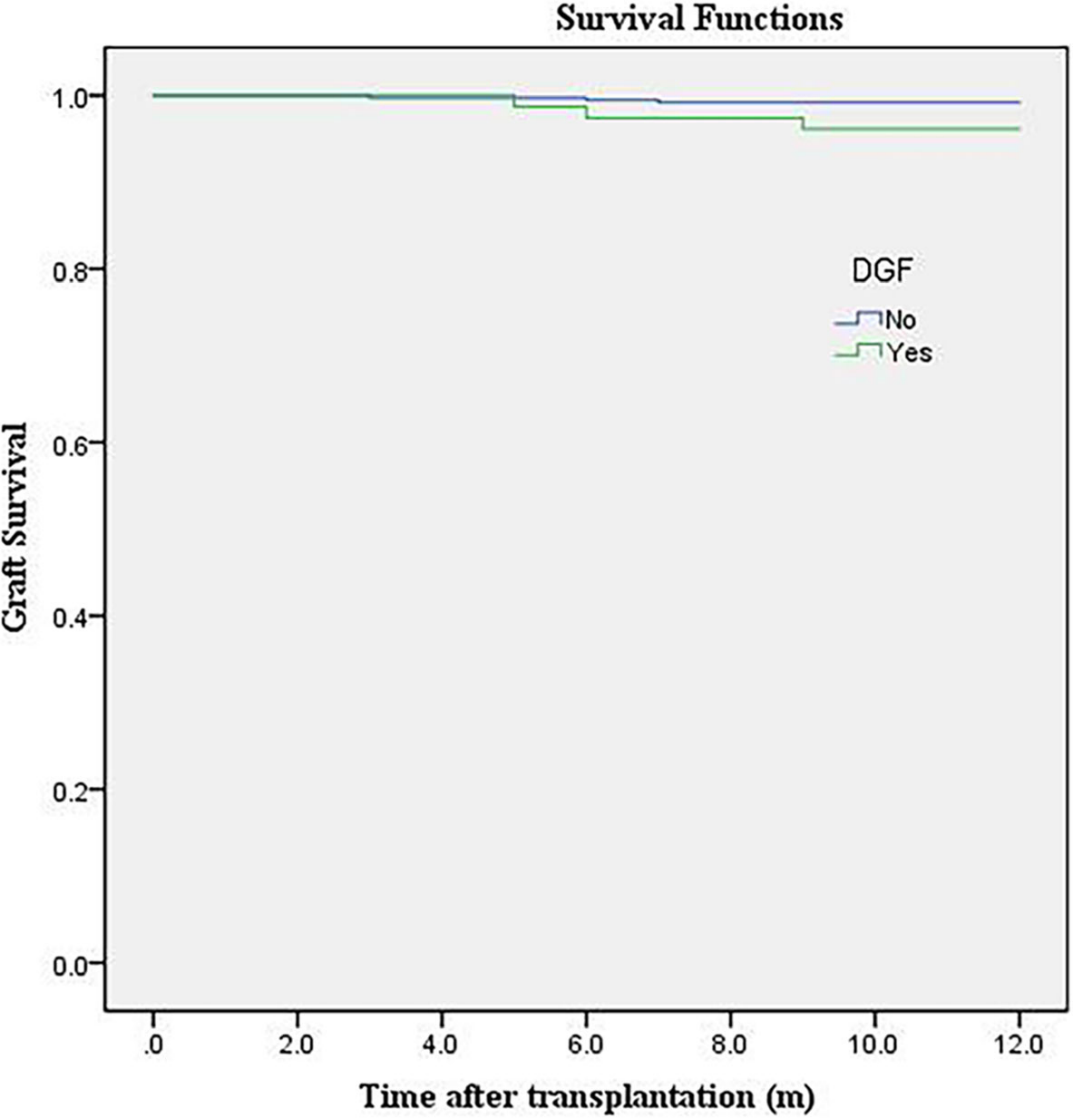
Kaplan–Meier plot of graft survival for DGF

Patients from the training cohort were observed every 2 weeks during the first postoperative year. We found that 5 DGF and 1 non-DGF patients experienced graft loss. The 1-year graft survival rates were 93.59 and 99.74%, respectively, for the groups with and without DGF. A Kaplan–Meier plot of graft survival against DGF occurrence is presented in Fig. 4. According to the log-rank test (*P* < 0.05), 1-year graft survival was impacted by DGF, though no correlation with KDPI based on the log-rank test was found (*P* > 0.05). Due to the limited number of graft losses (*n* = 6), Cox regression was not performed.

## Discussion

DGF is a common complication after kidney transplantation operations and is related to both short-term functional recovery and long-term survival of the transplanted kidney. Yarlagadda et al. [17] systematically reviewed the definitions of DGF, concluding that the combination of Scr reduction and dialysis needs constituted a reasonable definition. However, most centers have recently reached a consensus that the definition of DGF is the need for dialysis within the first week after transplantation [10–12]. As our study adopted the most common definition, DGF cases were relatively few. Consistent with the situation in America, the number of candidates waiting for kidney transplantation is increasing annually in China. Given the strain on kidney resources, the inclusion of ECD has been recognized internationally, even though the utilization of ECD is associated with increased cost and DGF [10]. A systematic review by Tingle et al. confirmed that machine perfusion reduced the incidence of DGF [18]; Patel et al. [19] also demonstrated that HMP can improve the utilization of kidneys. Thus, we used LifePort for marginal kidney perfusion to address such risk as much as possible. Despite the benefits of reducing the resistance parameter after perfusion, perfusion prolonged CIT; we chose 2 h as the perfusion time based on the research results of Patel et al. [18]. We discarded some bad kidneys according to the parameters of LifePort. Although the included marginal kidneys perfused by LifePort showed statistical correlation with DGF, we still used the kidneys that might have been discarded, accompanied by good 1-year graft survival.

Risk factors related to DGF can be divided into donor factors and recipient factors, and the multiple interactions of these factors ultimately affect the recovery of graft function. Numerous studies have assessed the causes of DGF, yet there is no consensus to date. In our study, prolonged CIT and WIT were most strongly associated with DGF, consistent with previous reports [20–27]. Additionally, a longer duration of recipient pretransplant dialysis was likely to lead to DGF. Other donor predictive factors included primary cause of death and terminal Scr. KDPI was introduced in America to indicate the quality of a kidney based on the data from OPTN. Zens et al. [14] also showed that a higher KDPI increased the rate of DGF for kidney recipients, and resulted in shorter graft survival. Nonetheless, other centers have suggested that a high KDPI is not a reason for rejecting a kidney because it does not result in a long-term mortality risk [15, 16]. In addition, a previous publication showed that the KDPI could not accurately predict pediatric donor kidney survival [28]. In our study, KDPI did not correlate with DGF and 1-year graft survival. The KDPI system does not include WIT and CIT, which may be one of the reasons for the difference. Additionally, the weight of each factor is fixed in the system; when the sample changes and decreases, accuracy also decreases.

Although immunity induction is an important step before surgery to avoid acute rejection, the use of ATG induction remains controversial. ATG may induce cytomegalovirus infections and hematological complications [29, 30]. Popat et al. [31] reported a lower DGF rate in the ATG-induced group among 45 patients in a single-center study. However, ATG induction did not reduce the risk of DGF in the research of de Sandes-Freitaseta [32]. In our study, the use of ATG depended on the patient’s economic condition and the surgeon’s preferences because it is costly, and this use was therefore not predictive. The results of our study showed that donor factors were the main influencing factors of DGF, likely because graft quality strongly affects renal function after transplantation. Terminal Scr is the most direct indicator of kidney quality. It is generally believed that the lower is the value, the lower is the incidence of DGF, as observed by Helfer [27] and in the present study.

Although long-term graft survival is expected, numerous complicated factors can cause graft loss. The relationship between DGF and deceased graft survival has been demonstrated recently [33, 34]. In a 3-year DCD kidney registry analysis, Lim et al. [34] reported that the recipients of DCD kidneys with DGF experienced a higher incidence of acute rejection and overall graft loss. Gill et al. [33] observed that the DGF-associated risk of graft failure was greatest in the first posttransplant year, and a meta-analysis by Yarlagadda et al. [35] verified the association between DGF, acute rejection, and graft survival. The present study performed patient follow-up in the training cohort for 1 year, with findings consistent with those reported by Gill et al.

Our nomogram is a simple and visual prediction model of posttransplant factors. Maier et al. investigated the relationship between DGF and posttransplant indicators of neutrophil gelatinase-associated lipocalin (NGAL), reporting that early assessment of serum and urinary NGAL could predict DGF [36]. In their retrospective cohort study, Cardinal et al. [37] used multivariate analysis to examine predictors of DGF but did not distinguish the importance of each. Irish et al. [3, 38] combined numerous donor and recipient factors, and applied nomogram scoring systems for predicting DGF in DD kidney transplantation, which were verified by ROC curves. Previous scoring systems are valuable; on this basis, we incorporated a certain percentage of marginal kidneys, especially young kidneys. Overall, young kidney transplantation was effective and safe, indicating a promising expansion of the donor pool. Infant donors younger than 5 months were excluded from this study, because our center used the novel method of en bloc kidney transplantation introduced by Dai et al. [39]. On the other hand, we investigated new indicators, including KDPI, LifePort and HCV history. The evaluation and therapeutic effects of LifePort on kidneys are worthy of affirmation, which is consistent with previous findings [40].

The present study applied a 10-fold cross-validation LASSO method to divide the data into 10 equal parts, with nine parts for the model and one part for validation. This process was repeated ten times to produce an accurate AUC.

In addition to identifying patients at higher risk of DGF before surgery, our model may also be used as a clinical tool to reduce the risk of DGF. CIT should be controlled when possible; for example, shortening the harvest and patient preoperative preparation times would reduce CIT, which would help to decrease the incidence of DGF. More specifically, the model can be used as a strategy to select suitable donors and recipients by identifying reasonable matches between recipients’ conditions and CIT. Additionally, the model can guide immunosuppression induction for high-risk DGF donors identified by the nomogram .

This study had some limitations. First, because this was a single-center study, the sample size was small and data were variable since. Second, the follow-up period of graft function was not long compared to 10–20 years. Finally, we did not provide solutions for predictors such as WIT. Future studies are needed to explore methods for shortening WIT and to investigate the factors influencing graft survival, as prolongation of graft survival is our ultimate aim.

## Conclusion

This study identified six risk factors as predictors of DGF, including donor CIT, WIT, terminal Scr, primary cause of death, and recipient duration of pretransplant dialysis. A visual nomogram with reliable accuracy was created for clinical use.

## Supplementary Information

**Additional file 1: Figure S1.** Flow diagram of patient screening.

**Additional file 2.** Supplement 2.

**Additional file 3.** Supplement 3.

## Data Availability

The data used in the study was extracted from our own database, and available from corresponding author on reasonable request.

## Abbreviations

DGF: Delayed graft function
CIT: Cold ischemia time
WIT: Warm ischemia time
BMI: Body mass index
DD: Deceased donation (DD)
DBD: Donation after brain death
DCD: Donation after cardiac death
ATN: Acute tubular necrosis
IGF: Immediate graft function
SGF: Slow graft function
ECD: Expanded criteria donor
OPTN: Organ Procurement and Transplantation Network
Scr: Serum creatinine
HPM: Hypothermic machine perfusion
ATG: Antithymocyte globulin
IL-2: Interleukin 2
ICU: Intensive care unit
CPR: Cardiopulmonary resuscitation
HCV: Hepatitis C virus
KDPI: Kidney donor profile index
HLA: Human leukocyte antigen
PRA: Plasma renin activity
AUC: Area under the receiver operating characteristic curve
LASSO: The Least absolute shrinkage and selection operator
SD: Standard deviation
CI: Confidence interval
NGAL: Neutrophil gelatinase-associated lipocalin
