# Supplementary figures and images for: Predictors and one-year outcomes of patients with delayed graft function after deceased donor kidney transplantation
Source: BMC Nephrol. 2020 Dec 4;21:526. doi: 10.1186/s12882-020-02181-1 (PMC7716446; doi:10.1186/s12882-020-02181-1)

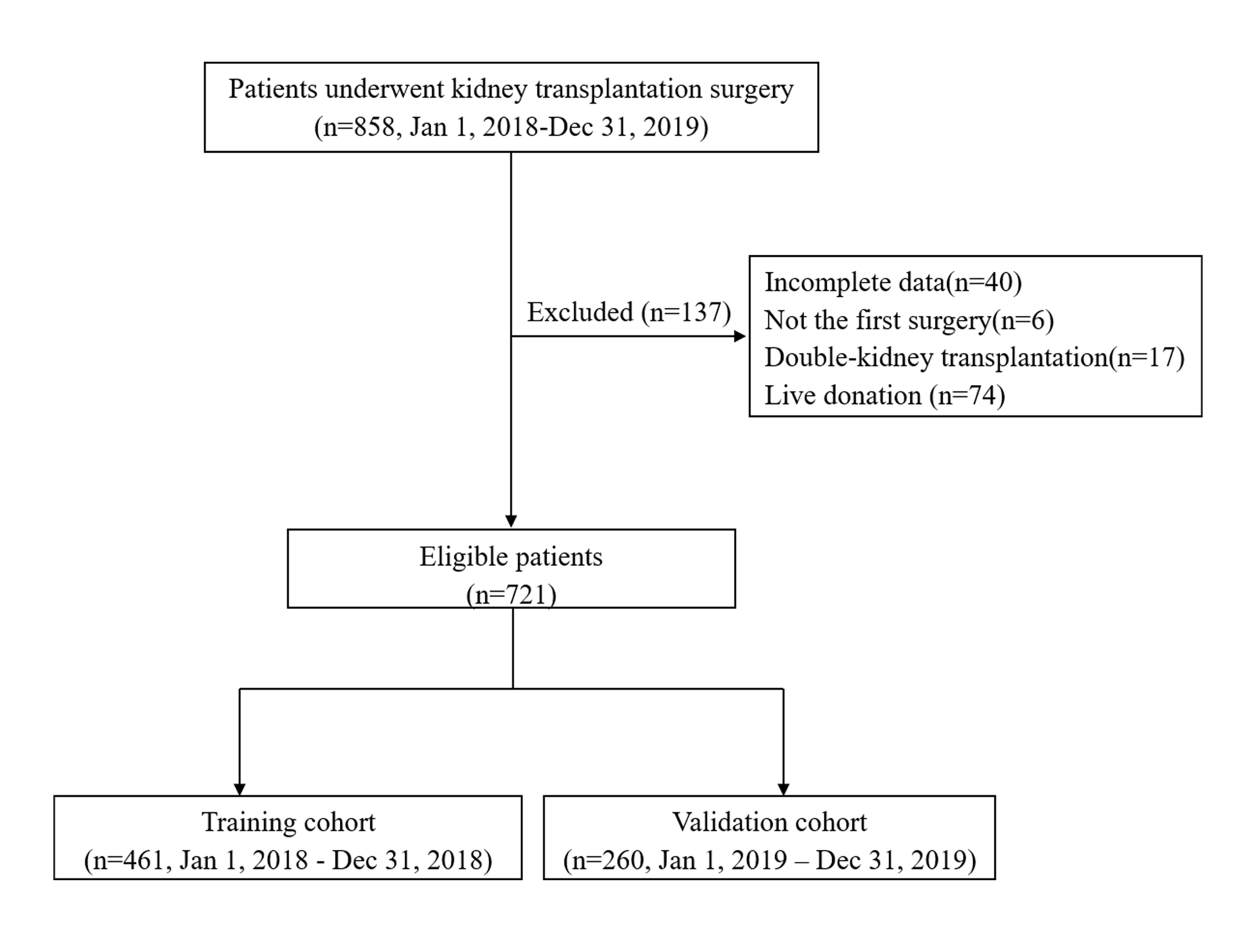

Supplement: Supplementary file 1 — Additional file 1: Figure S1. Flow diagram of patient screening. [file 12882_2020_2181_MOESM1_ESM.tif]
