# Supplementary material for: Predictors and one-year outcomes of patients with delayed graft function after deceased donor kidney transplantation
Source: BMC Nephrol. 2020 Dec 4;21:526. doi: 10.1186/s12882-020-02181-1 (PMC7716446; doi:10.1186/s12882-020-02181-1)
Supplement: Supplementary file 2 — Additional file 2. Supplement 2. [file 12882_2020_2181_MOESM2_ESM.pdf]

```
library(foreign)
library(rms)
options(datadist = 'ddist')

tab<-read.table("D:/Administrator/Documents/10_05.csv", sep=',')
tab<-as.data.frame(tab)
head(tab)

# CITWIT  Dialysis duraton       Terminal Scr     Primary cause Lifeport    DGF
colnames(tab) = c('CIT', 'WIT', 'Dialysis duration', 'Terminal Scr', 'Primary.cause', 'Lifeport', 'DGF')

tab[,1]<-as.numeric(tab$CIT)
tab[,2]<-as.numeric(tab$WIT)
tab[,3]<-as.numeric(tab$`Dialysis duration`)
tab[,4]<-as.numeric(tab$`Terminal Scr`)
tab[,5]<-factor(tab$`Primary.cause`)
tab[,6]<-tab$`Lifeport`
tab[,7]<-as.numeric(tab$`DGF`)


ddist <- datadist(tab)

f <- lrm(DGF~., data=tab, x= T, y=T)

nom1 <- nomogram(f, fun=function(x) 1/(1+exp(-x)),
                        funlabel = 'Risk of DGF',
                        fun.at = c (0.05, seq (0,1, by=0.1),0.95))

nom1

#plot(nom1,
#        col.grid = gray(c(0.90,0.95)))
```
