# Supplementary material for: Predictors and one-year outcomes of patients with delayed graft function after deceased donor kidney transplantation
Source: BMC Nephrol. 2020 Dec 4;21:526. doi: 10.1186/s12882-020-02181-1 (PMC7716446; doi:10.1186/s12882-020-02181-1)
Supplement: Supplementary file 3 — Additional file 3. Supplement 3. [file 12882_2020_2181_MOESM3_ESM.pdf]

Score of CIT

CIT (hours)	Score
2	0
4	2
6	5
8	7
10	9
12	12
14	14
16	16
18	19
20	21
22	23
24	26

Score of WIT

WIT (minutes)	Score
0	0
2	8
4	15
6	23
8	31
10	38
12	46
14	54
16	62
18	69
20	77
22	85
24	92
26	100

Score of dialysis duration

Duration (months)	Score
0	0
20	3
40	6
60	9
80	12
100	15
120	18
140	20
160	23
180	26
200	29

Score of terminal Scr

Scr (mg/dL)	Score
0.0	0
0.5	5
1.0	9
1.5	14
2.0	18
2.5	23
3.0	27
3.5	32
4.0	36
4.5	41

Score of primary of cause

Category	Score
Head trauma	0
Stroke	39
Other	39

Score of Lifeport perfusion

Category	Score
No	0
Yes	25

Points per unit of linear predictor: 13.42841

Linear predictor units per point: 0.07446896

Total Score of DGF

Total score	Risk of DGF
50	5%
60	10%
71	20%
78	30%
84	40%
89	50%
95	60%
101	70%
108	80%
119	90%
129	95%
